# Eyeblink rate watching classical Hollywood and post-classical MTV editing styles, in media and non-media professionals

**DOI:** 10.1038/srep43267

**Published:** 2017-02-21

**Authors:** Celia Andreu-Sánchez, Miguel Ángel Martín-Pascual, Agnès Gruart, José María Delgado-García

**Affiliations:** 1 Neuro-Com Research Group, Universitat Autònoma de Barcelona, Barcelona, Spain; 2 Division of Neurosciences, Pablo de Olavide University, Seville, Spain

## Abstract

While movie edition creates a discontinuity in audio-visual works for narrative and economy-of-storytelling reasons, eyeblink creates a discontinuity in visual perception for protective and cognitive reasons. We were interested in analyzing eyeblink rate linked to cinematographic edition styles. We created three video stimuli with different editing styles and analyzed spontaneous blink rate in participants (N = 40). We were also interested in looking for different perceptive patterns in blink rate related to media professionalization. For that, of our participants, half (n = 20) were media professionals, and the other half were not. According to our results, MTV editing style inhibits eyeblinks more than Hollywood style and one-shot style. More interestingly, we obtained differences in visual perception related to media professionalization: we found that media professionals inhibit eyeblink rate substantially compared with non-media professionals, in any style of audio-visual edition.

Narrative fragmentations in media are reflected through edition. Cinematographic edition was soon considered a generator of connections, ideas, and contents. By joining two shots, new content is created. This surprising result shows the mechanisms organizing our attention and perception of cinematographic phenomena^1^. In some contexts, filmic edition is related to the audio-visual narrative itself and editing rules; on other occasions, cuts in the narration give the narrator the possibility of saving time and space, moving faster in the story. In this article, we study the relation between audio-visual editing styles and the viewer’s eyeblink rate. The research also deals with the role of media professionalization in this context.

## Evolution of editing styles

The initial analysis of edition is usually ascribed to the 1910 s and the beginning of Hollywood’s Golden Age (with D.W. Griffith^2^,^3^) and the Soviet school of cinema. Kuleshov’s^4^ and Pudovkin’s^5^ experiments, and Eisenstein’s^6^ filmic and written work, are exponents of analytic and metaphoric edition, as well as of visual conflict.

As the history of cinema moved along, so did rules and patterns in edition. According to Burch^7^, in the passage from Primitive Mode of Representation (PMR) to Institutional Mode of Representation (IMR), audio-visual language was equipped with details of the scene and was released from the constrictions of theatrical narrative, continuity being effected through editing rules. Such is the importance of the IMR edition system that continuity editing rules are known by the name of the whole: Hollywood style^3^,^8^. Basically, Hollywood style’s rules are based on the relationships between content, rhythm, space, and time in two shots that are linked: high use of general-situation shots, smooth transitions between shots, rhythm in edition guided by shot size, continuity in perceptive space (180° rule), and use of transitions for ellipsis^3^,^9^.

Later, a more intensified edition appeared, known as post-classical style, but which could also be named MTV style because of its extended use in musical video clips. This form included an intensified continuity with faster edition, a distinctive use of extreme focal-length lenses, tight framings, and more movement in camerawork^10^,^11^.

## Understanding film stories, despite cuts

As the filmic language has advanced, multiple prescriptions or “educational” standards among the various styles of audio-visual edition have been discussed. For instance, Dmytryk^12^ made some interesting proposals about media edition: not to cut a shot without a reason, to cut in internal movement of actors, the need to start and end the scenes with actions, and—above all—content as a priority over aesthetics. He also talked about the viewer’s eyeblink as a sign of distraction to be taken into account^12^. This has also been stressed by the editor Walter Murch^13^, who, after working in Hollywood for several years on films such as *The Godfather* (1972) or *Apocalypse Now* (1979), suspected that eyeblink could have a comprehension function in films. He wondered whether there is a predictable and measurable blink that could let him know the best moment to cut a shot. Previously and in parallel, from fields such as psychology and frequently from research detecting failures in human communication, eyeblink has been understood as a reflection of attention and cognitive activity^14^,^15^,^16^. Despite eyeblinks, with their loss of visual input, visual experience is maintained^17^, and despite the cuts in a movie, narrative content continues being understood.

Nakano and colleagues^18^ have shown synchronization in eyeblinks between and within subjects watching short video stories. Interestingly, viewer’s eyeblinks, connected to comprehension^19^, seem to be linked not to cuts^18^,^20^ but to narrative. This leads us to suspect that cuts respecting continuity editing rules would not affect narrative comprehension. In 2010, Schwan and Ildirar showed audio-visual fragments with different editing styles and perspectives to isolated people living in the Turkish mountains, who had never before seen an audio-visual work^21^,^22^. All participants in that experiment understood that what they were watching was a movie, and none of them confused it with reality. Edition kept viewers from confusing screened presentation with reality. Separate shots were understood, but narrative structure was not integrated continuously through the work. Participants also did not perceive the subjective camera as the viewer’s angle. Moreover, they never understood that the camera could become a substitution of the observer’s view. Some cinematographic discontinuity techniques construct an apparent continuum from reality to abstraction, but previous experience is required for its comprehension^21^. Continuity seems to be related to the spectator’s expectation and attention but not to stimulus characteristics^23^,^24^,^25^. So, perceptive continuity in our real world seems to be fundamental for the understanding of ellipsis in edition.

## Understanding film stories, despite eyeblinks

We all assume that if we close our eyes, the world will still be there when we open them. Eyeblink hides visual flow for between 150 and 400 milliseconds^26^,^27^,^28^,^29^. Thus, visual information is hidden from viewers. Blinks interrupt the viewer’s visual perception and are linked not only to corneal wetting and protection, but also to attention and narrative comprehension^18^,^19^,^20^,^29^. In an audio-visual context, during a conversation eyeblinks are synchronized between listener(s) and talker^30^ by narrative pauses of speech. The impact of continuity editing on how people perceive events in a narrative film has been studied in the context of brain networks^31^. However, there are no significant differences in eyeblinks within editing boundaries^20^.

Eyeblink while viewing video scenes has also been related to different empathy and engagement in Autism Spectrum Disorders (ASD)^32^. Also, it has been observed while watching videos, that eyeblink is connected with attentional processes, momentarily decreasing cortical activity in the dorsal attention network, but increasing at the same time the—sometimes questioned—Default Mode Network (DMN), involved in introspection processes, when the brain is not engaged in concrete tasks^33^. Likewise, it has been connected to audio-visual consumption via screens^27^,^34^,^35^,^36^. The correlation and synchronization in cognitive states in subjects watching films have also been studied^30^,^37^. And there seems to be a synchrony between eyeblink and the interest in the story^18^. With those previous studies, we suspected that eyeblinks may be different depending on the attention given to video works by media and non-media professionals. Recently, the effects of camera movement^38^ and the impact of continuity in cinematographic editing^31^,^39^,^40^ have been analyzed from a cognitive perspective. It has also been proposed that event segmentation is automatic and depends on processing meaningful changes in the perceived situation^41^. These previous investigations made us think that editing style could also affect eyeblinks.

## Professionalization

Previous studies have indicated different cognitive patterns linked to professionalization in, for example, musicians^42^, surgeons^43^, athletes^44^, or drivers^45^,^46^. Wong and colleagues^43^ noted that the rate in ophthalmologic surgeons decreased during the execution of surgery, from 16 to 4 eyeblinks per minute. Because of this professionalization effect, and given the vision and attention-related job of media professionals, we expect that media professionalization affects visual behavior. Here, we consider the existence of cognitive differences between media professionals and non-media professionals.

The present investigation addresses two aims. First, to know differences in eyeblink rate according to editing styles; and, second, to find out whether the different editing styles evoke similar patterns of eyeblink rate in media professionals and non-media professionals. The results suggest the presence of specific visual cognitive patterns in media professionalization.

## Results

### Descriptive results

**Figure 1:**
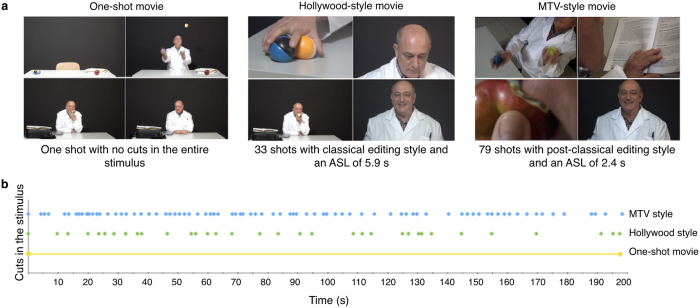
Video stimuli with different editing style. (**a**) Three editing styles were used for the video stimuli creation. The one-shot movie comprised a single open shot where the character was always positioned in the middle of the picture. The Hollywood-style edited movie comprised different classical shots such as close-ups, medium shots, and open shots, following a classical editing style. It was created from 33 shots with an ASL of 5.9 seconds. The MTV-style edited movie was created from 79 shots with an ASL of 2.4 seconds. It had close-ups, big close-ups, full shots, medium shots, open shots, aerial shots, reverse shots, low-angle shots, no time and space continuity, different angles, and a much less homogeneous edition than classical style. (**b**) Each dot represents a cut in the edited movies. The one-shot movie had no cuts in the visual narrative; the Hollywood- and MTV-style movies had repeated cuts. Those cuts were distributed along the videos, as represented by the dots.

**Figure 2:**
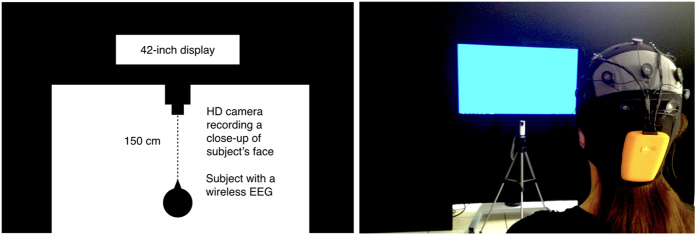
Experimental set-up. The video stimuli were presented on a 42-inch display at a distance of 150 cm from the subject. We recorded the participant’s eye movements with an HD camera, and the EMG activity of the orbicularis oculi muscle with a wireless EEG/EMG device.

**Figure 3:**
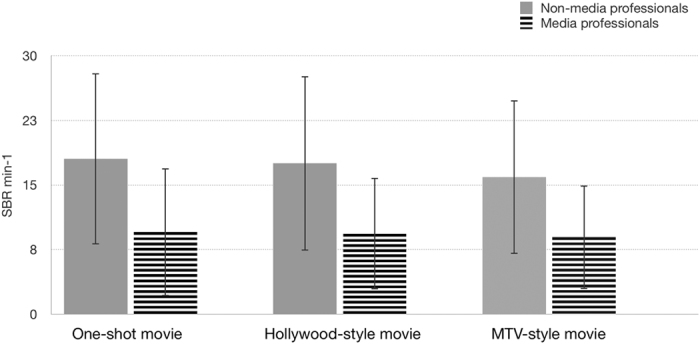
Comparison of spontaneous blink rate (SBR) in non-media professionals and media professionals in different editing styles. According to our results, non-media professionals have a higher SBR than media professionals in every editing-style movie; this may be due to the occupational use of screens by media workers. Our results suggest that the shorter the length of the shots in an audio-visual work, the lower the SBR. Our data show a greater homogeneity in media professional subjects than in non-media professionals in every editing style analyzed: one-shot movie (non-media professionals, SD = ±9.338; media professionals, SD = ±7.386), Hollywood-style movie (non-media professionals, SD = ±10.108; media professionals, SD = ±6.462), and MTV-style movie (non-media professionals, SD = ±8.899; media professionals, SD = ±6.012).

Participants were presented different editing-style stimuli with different Average Shot Length (ASL): i) a one-shot movie (with no cuts), ii) a Hollywood-style movie (ASL = 5.9 sec), and iii) an MTV-style movie (ASL = 2.4 sec), but all with the same narrative (Fig. 1). Spontaneous Blink Rate (SBR) of the subjects was recorded with an electromyographic (EMG) wireless system and a High-Definition (HD) camera (Fig. 2). The mean SBR (N = 40) was 13.776 min^−1^ (SD = ±9.641) in the one-shot movie; 13.427 min^−1^ (SD = ±9.338) in the Hollywood-style movie; and 12.421 min^−1^ (SD = ±8.283) in the MTV-style movie. However, large differences were found between non-media professionals (n = 20) and media professionals (n = 20) in every stimulus (Fig. 3): the one-shot movie evoked a mean SBR of 18.018 min^−1^ (SD = ±9.917) in non-media professionals and of 9.534 min^−1^ (SD = ±7.386) in media professionals, the effect size, d = −0.97 and r = −0.436, exceeded Cohen’s^47^ convention for a large effect (d = 0.80); the Hollywood-style movie evoked a mean SBR of 17.508 min^−1^ (SD = ±10.108) in non-media professionals and of 9.347 min^−1^ (SD = ±6.462) in media professionals, with an effect size of d = −0.962 and r = −0.433; and the MTV-style movie 15.9 min^−1^ (SD = ±8.899) in non-media professionals and 8.941 min^−1^ (SD = ±6.012) in media professionals, with an effect size of d = −0.916 and r = −0.416. According to these descriptive results, the order in the frequency of evoked SBR depending on the style of movie edition is (from lower to higher): MTV style – Hollywood style – one-shot style. This means that the lower the ASL a film’s cuts have, the lower the SBR its viewers present. Our descriptive results also show clear differences in SBR related to media professionalization. We carefully analyzed all these results, looking for significant statistical differences.

### Differences in eyeblink based on editing style: one-shot style, Hollywood style and MTV style

The statistical comparison of the frequency of the eyeblinks evoked by the different movie editing styles, independently of the professional status, showed significant differences between them (*X*^2^(2) = 7.2, *p* = 0.027, Friedman Test, non-parametric test). Pairwise comparison showed a substantial statistical difference only between one-shot movie and MTV-style movie (*p* < 0.05, Tukey post hoc Test for Friedman Test, Dunn post hoc Test for Friedman Test, and Student-Newman-Keuls post hoc Test for Friedman Test). By groups, we obtained highly significant differences between editing styles in non-media professionals (*p* < 0.01), (*X*^*2*^(2) = 11.1, p = 0.004, Friedman Test, non-parametric test). We did, again, pairwise comparison between the different editing styles. We obtained substantial differences between one-shot movie and MTV style (*p* < 0.05, Tukey post hoc Test for Friedman Test, Dunn post hoc Test for Friedman Test and Student-Newman-Keuls post hoc Test for Friedman Test) and, also, between one-shot movie and Hollywood style, on one hand, and Hollywood style and MTV style, on the other (*p* < 0.05, Student-Newman-Keuls post hoc Test for Friedman Test. However, media professionals did not show significant differences in eyeblink rate based on editing styles (*F* (2, 19) = 0.487, *p* = 0.618, one-way Repeated Measures ANOVA).

With the aim of contrasting these results, we also did a pairwise comparison (one-shot movie – Hollywood-style movie; Hollywood-style movie – MTV-style movie; one-shot movie – MTV-style movie) (Table 1). Again, we obtained significant differences between one-shot movie and MTV style in the whole group (*W* = −358, *Z* = −2.406, *p* = 0.016, Wilcoxon Signed Rank Test, non-parametric test), and specifically in non-media professionals (*W* = −132, *Z* = −2.464, *p* = 0.012, Wilcoxon Signed Rank Test, non-parametric test). The rest of the pairwise comparisons did not show significant differences.

According to our results, MTV editing style inhibits eyeblink rate considerably, compared with one-shot movie edition. However, this is not significant in media professionals. Non-media professionals also show substantial (but not so strong) differences in SBR between Hollywood and MTV styles, and between one-shot movie and Hollywood style. Interestingly, media professionals do not change their blink rate depending on the style of the edition, while non-media professionals do.

### Differences in eyeblink rate between non-media and media professionals

Several studies have shown cognitive differences related to professionalization in many areas, as mentioned before^43^,^44^,^45^,^46^,^47^. Here we found that, on the whole, there are statistical differences in eyeblink rate between these two groups (*p* < 0.01), (Mann-Whitney *U* = 86, n = 20, *p* = 0.002, Mann-Whitney Rank Sum Test, non-parametric test). These differences are present in every edited movie style analyzed: one-shot movie (*p* < 0.01), (Mann-Whitney *U* = 86.5, n = 20, *p* = 0.002, Mann-Whitney Rank Sum Test, non-parametric test); Hollywood-style movie (*p* < 0.01), (Mann-Whitney *U* = 90, n = 20, *p* = 0.003, Mann-Whitney Rank Sum Test, non-parametric test); and MTV-editing-style movie (*p* < 0.01), Mann-Whitney *U* = 94.5, n = 20, *p* = 0.004, Mann-Whitney Rank Sum Test, non-parametric test).

In accordance with the results, there is a professionalization of visual perception, related to eyeblink rate, in media workers. They show statistical differences in each type of editing style, with lower SBR than non-media professionals.

## Discussion

Differences in the style of edition of an audio-visual work have consequences for the viewers’ visual perception. The eyeblink rate decreases with the decrease of the ASL. This may be to lessen the effect of the discontinuity created by the constant cuts. In our results, the MTV-editing-style movie evoked the lowest eyeblink rate, followed by the Hollywood style, and, finally, the one-shot style. These differences were statistically significant when comparing MTV style with a one-shot movie, but no substantial differences were found comparing Hollywood style with MTV style. This may be due to the length of the stimuli used (198 seconds). Probably, longer stimulus duration would have increased differences between these two edition styles. We decided not to use such stimuli in our study in an attempt to avoid participants’ boredom during experimentation sessions. However, in the future, we plan to test this hypothesis with different and longer stimuli. Attention has been reported to be connected with the frequency of eyeblink^18^. Thus, according to our results, an MTV editing style should increase the attention level of viewers, though we did not apply an attention-level protocol to determine and confirm this. By groups, while non-media participants showed differences in SBR between each style of edition, media professionals did not. They had a similar eyeblink rate, independently of the ASL.

We suggest that this result may be a consequence of the high level of attention they are used to paying to audio-visual tasks as part of their job. It seems that in their viewing, attention level is more important than the characteristics of the media stimuli.

We found big differences in eyeblink rate between non-media and media professionals in every editing style analyzed. The former presented a higher frequency of eyeblink than the latter, regardless of the style of edition. This could be of interest for media professionals’ visual health. Our results, then, suggest that there are differences in visual perception between these two groups, and that media professionalization decreases eyeblink rate, independently of the media work characteristics. Eyeblinks also have physiological functions^48^, since they protect the cornea and participate in the visual process^49^,^50^. An excessive decrease of eyeblink as a result of spending many hours using screens can provoke computer vision syndrome^35^, including corneal lesions, blurred vision, or dry eyes. Thus, editing styles linked to an increase in eyeblink rate should be of interest in audio-visual production contexts and to departments of labor charged with workers’ safety and health. Making longer shots (with higher ASL) in audio-visual works could help to prevent visual problems for viewers. However, this could also affect their attention. Therefore, media creators should balance these two parameters (eye protection and attention) for the sake of their viewers and of their own success. These results invite us to continue with research to find cognitive patterns in media professionalization. At the same time, we wonder whether different editing styles might also show differences related to the number of hours of media consumption. It would be interesting to compare young people, who spend a lot of time watching media content, with media professionals. We think that media professionalization might also be a factor in that comparison.

## Methods

### Participants

Twenty media professionals (16 male, 4 female; age 30–56 years) and 20 non-media professionals (15 male, 5 female; age 28–56 years) with normal or corrected-to-normal visual acuity took part in the study. Media professionals were chosen following the criterion of a minimum of 6 years of experience making decisions related to media editing and audio-visual cuts in their everyday work. They were producers, assistant producers, cameramen, image controllers, documentalists, graphic designers, post-production editors, sports commentators, and video editors. Non-media professionals were carefully chosen outside this criterion, being individuals who did not make decisions related to media editing and audio-visual cuts in their work. They were journalists without media editing responsibilities, computer specialists, administrative and management assistants, telecommunication engineers, electronics technicians, stylists, specialists in prevention of occupational risks, and executive producers without artistic profile. All experiments were performed in accordance with relevant guidelines and regulations, with the ethics approval of the University Autònoma de Barcelona (Ethics Commission for Research with Animals and Humans, CEEAH); all participants (N = 40) gave prior written informed consent.

### Experimental stimuli

Four stimuli with the same narrative but different video edition style were designed (Fig. 1). Three of them were videos of 198 seconds, and the fourth was a live enactment of around the same duration. The first stimulus was a one-shot movie with an open shot of the action. The second stimulus was edited according to IMR or classical Hollywood style with a total of 33 different shots and an ASL of 5.9 seconds. The third stimulus was edited by post-classical or MTV-style rules; it comprised 79 shots that did not follow classical editing rules, and had an ASL of 2.4 seconds. The fourth stimulus was a live enactment of the same audio-visual storytelling. The four stimuli were presented preceded by 30 seconds of black screen. The narrative in all the videos and the enactment was the same: a man entered a room, sat at a desk, juggled with three balls, opened a laptop, looked up information in some books, wrote in the laptop, closed the laptop, ate an apple, looked directly into camera with alternate friendly and sad expression, and left the room (Supplementary Video S1).

### Experimental set-up

The video stimuli were presented on a 42-inch HD LED display (PanasonicTH-42PZ70EA, Panasonic Corporation). Participants were placed 150 cm in front of the screen. The stimuli were presented and synchronized with Paradigm Stimulus Presentation (Perception Research System Incorporated), and stimulus presentation was randomized.

Participants’ faces were recorded throughout in a close-up shot, with an HD video camera (Sony HDR-GW55VE, Sony Corporation) at 25 frames per second. Simultaneously, EMG signals were recorded with the help of a wireless Enobio 20 EEG/EMG system (Neuroelectrics^®^) and of NicOffline software (Neuroelectrics^®^) for data acquisition. The EMG electrodes were placed according to the international 10–20 system. The experimental set-up allowed participants to be comfortable during the sessions (Fig. 2).

### Data analysis

For the analysis of the data, we used results from the three video stimuli. We analyzed eyeblinks following two methods: EMG signals and recorded HD videos. For the EMG signal, we worked with Brainstorm^51^, filtered to 0.5–3 Hz, and applied Brainstorm’s eyeblink detectors in Electrooculogram, Fp1 and Fp2. Then, we carefully checked the detections obtained, as proposed by Tadel and colleagues^52^. For the recorded HD videos, we manually looked for the eyeblinks in each participant and stimulus, following the guidelines of Nakano and colleagues^18^. We compared one by one results obtained from the two methods to check and get a final list of eyeblinks for later analysis. Statistical analysis was carried out with Sigmaplot 11.0 (Systat Software Inc.) with parametric and non-parametric tests based on Shapiro-Wilk Test results for testing the normality of the data. We used an alpha level of 0.05 for all statistical tests. We worked with the mean of eyeblinks per minute in each stimulus.

## Additional Information

**How to cite this article**: Andreu-Sánchez, C. *et al*. Eyeblink rate watching classical Hollywood and post-classical MTV editing styles, in media and non-media professionals. *Sci. Rep.* **7**, 43267; doi: 10.1038/srep43267 (2017).

**Publisher's note:** Springer Nature remains neutral with regard to jurisdictional claims in published maps and institutional affiliations.

## Supplementary Material

Supplementary Video S1

Supplementary Information

## Figures and Tables

**Table 1:** Pairwise comparisons in editing styles.

Editing Styles: Pairwise Comparison		p-value	
One-shot movie – *Hollywood-style*	Z = −1.29	0.199	Wilcoxon Signed Rank Test
One-shot movie – *Hollywood-style*, in non-media professionals	t(19) = 0.704	0.490	t-test
One-shot movie – *Hollywood-style*, in media professionals	t(19) = 0.272	0.789	t-test
*Hollywood-style* – *MTV-style*	Z = −1.909	0.057	Wilcoxon Signed Rank Test
*Hollywood-style* – *MTV-style*, in non-media professionals	t(19) = 1.760	0.094	t-test
*Hollywood-style* – *MTV-style*, in media professionals	t(19) = 0.764	0.454	t-test
One-shot movie – *MTV-style*	Z = −2.406	0.016*	Wilcoxon Signed Rank Test
One-shot movie – *MTV-style*, in non-media professionals	Z = −2.464	0.012*	Wilcoxon Signed Rank Test
One-shot movie – *MTV-style*, in media professionals	t(19) = 0.969	0.345	t-test

Results from pairwise comparison between the editing styles analyzed in this investigation: one-shot, Hollywood–style, and MTV-style. We applied a Wilcoxon Signed Rank Test for non-normal distributions, and a two-tailed t-test for normal distributions. Previously, we used a Normality Test (Shapiro-Wilk) for analyzing normality distribution of data. We found substantial differences between one-shot movie and MTV editing styles for the whole set of subjects, and for the group of non-media professionals. *p < 0.05.
