# Supplementary material for: Eyeblink rate watching classical Hollywood and post-classical MTV editing styles, in media and non-media professionals
Source: Sci Rep. 2017 Feb 21;7:43267. doi: 10.1038/srep43267 (PMC5318946; doi:10.1038/srep43267)
Supplement: Supplementary Information [file srep43267-s2.pdf]

Scientific Reports
Supplementary Video

Eyeblink rate watching classical Hollywood and post-classical MTV editing styles, in media and non-media professionals

Celia Andreu-Sánchez[1]*, Miguel Ángel Martín-Pascual[1], Agnès Gruart[2] and José María Delgado-García[2]

[1] Neuro-Com Research Group, Universitat Autònoma de Barcelona, Barcelona, Spain.

[2] Division of Neurosciences, Pablo de Olavide University, Seville, Spain.

*** Corresponding author**
Dr. Celia Andreu-Sánchez
Neuro-Com Research Group
Universitat Autònoma de Barcelona
Barcelona, Spain
Phone: 0034 93 581 4463
E-mail: celia.andreu@uab.cat

Supplementary Information

Supplementary Video S1. Stimulus designs for one-shot movie, classical Hollywood-style movie, and modern MTV-style movie. Fragments of the 3 video stimuli used in this experiment: 20 seconds of the one-shot style movie, 20 seconds of the Hollywood-style movie, and 20 seconds of the MTV-style edited movie.
